# DNA methylation profiles of ovarian cysts resemble ovarian tissues but not endometrial tissues

**DOI:** 10.1186/s13048-024-01440-1

**Published:** 2024-06-06

**Authors:** Xiaohui Zhang, Xiaojing Zhao, Jiapo Wang, Yifang Zhang, Jinhong Chen

**Affiliations:** 1 Shanghai First Maternity and Infant Hospital, TongJi University School of Medicine, No. 2699, hi-tech west road, Pudong new area, Shanghai, 201204 China; 2 School of Life Science and Technology, ShanghaiTech University, Shanghai, 201204 China; 3 Changxing People’s Hospital of Zhejiang Province, Zhejiang, 313100 China

**Keywords:** Ovarian endometriosis, DNA methylation, Tissue of origin

## Abstract

**Introduction:**

Endometriosis is a heritable, complex chronic inflammatory disease, for which much of the causal pathogenic mechanism remain unknown.Despite the high prevalence of ovarian chocolate cyst, its origin is still under debate.

**Methods:**

Prevailing retrograde menstruation model predicts that ectopic endometrial cells migrate and develop into ovarian chocolate cyst. However, other models were also proposed. Genome-wide association studies (GWASs) have proved successful in identifying common genetic variants of moderate effects for various complex diseases.

**Results:**

A growing body of evidence shows that the remodeling of retrograde endometrial tissues to the ectopic endometriotic lesions involves multiple epigenetic alterations, such as DNA methylation, histone modification, and microRNA expression.Because DNA methylation states exhibit a tissue specific pattern, we profiled the DNA methylation for ovarian cysts and paired eutopic endometrial and ovarian tissues from four patients. Surprisingly, DNA methylation profiles showed the ovarian cysts were closely grouped with normal ovarian but not endometrial tissues.

**Conclusions:**

These results suggested alterative origin of ovarian cysts or strong epigenetic reprogramming of infiltrating endometrial cells after seeding the ovarian tissue. The data provide contributing to the pathogenesis and pathophysiology of endometriosis.

**Supplementary Information:**

The online version contains supplementary material available at 10.1186/s13048-024-01440-1.

## Background

Ovarian endometriosis has a high prevalence and affects millions of women worldwide. The origin of endometriosis is still puzzling. It is generally considered to be derived from endometrium [1], however, other hypotheses were also provided. For instance, a latest report suggested that ~ 60% of endometriosis may originate from fallopian tube [2]. Ovarian origin recently, researchers found and confirmed the existence of cells expressing two germ cell line specific proteins (deadboxpolypeptide4/interferoninducedtrans, membraneprotein3, ddx4/ifitm3) in ectopic ovarian EMS lesions, suggesting that ovary-originated stem cell may contribute to the occurrence and progression of ovary EMS lesions [3]. To answer this question, we resorted to paired DNA methylation assay for ovarian endometriosis(ovarian endometrioma), normal ovarian and endometrial tissues in a small pilot cohort.

Epigenetic modifications regulate the transcriptional process, and abnormalities lead to various diseases. Among the most investigated and vital modifications, DNA methylation was linked to alterations in endometrial function/dysfunction-related genes, modulating cell proliferation, inflammation/immune response, angiogenesis, and steroid hormone response. These findings offer a deep understanding of epigenetic reprogramming and steroid hormone effects in endometrium aiding in the pathogenesis and pathophysiology of endometriosis [4–6].

Previous reports have shown that the endometriosis pathophysiology involves aberrant promoter methylation of several genes, for instance, HOXA10 [7], steroidogenic factor 1 [8] and aromatase [9]. As a new research area, genome-wide DNA methylation analysis can clarify the relationship between DNA methylation state and endometriosis. Our study utilized a microarray-based approach to determine the accurate DNA methylation level. The abnormal methylation expression between ovarian endometriosis cells, eutopic endometrium and ovarian tissues was analyzed.

## Methods

### Sample collection

Endometrium was biopsied from 4 fertile women receiving surgery for histologically confirmed unilocular/multilocular ovarian endometriosis (2:2). Normal ovarian tissue and eutopic endometrium of the same patient were used as controls. Specimens were obtained from subjects without hormone-based treatments at least 1 quarter before specimen acquisition and menstruation just clean, that is, endometrial hyperplasia. Upon surgical dissection, partial tissue was cryopreserved for microarray analyses. Endometrium and cysts were rinsed by Dulbecco’s modified eagle’s medium (DMEM) comprising of glutamine, 50 mg/ml streptomycin, and 50iu/ml penicillin (all from Invitrogen, Paisley, UK), and subsequently cut to a size of 1mm^3^. Then, ESC was isolated via screening with 70 mm nylon mesh after 2 h of collagenase (Sigma, Mo, USA) procession in a shaking incubator at 37℃. The filtrate was washed three times. The human investigation committee of Tongji University endorsed the research.

### DNA methylation analysis

Illumina 850k methylation EPIC Bead Array data was processed through a standard pipeline using the ChAMP package in R. The ChAMP can load raw intensity data in the form of IDAT or matrix containing methylation value (beta) of each probe [10]. After filtering and normalization, the 12 samples were combined into an array including only overlapping CpG sites and finally 743,800 probes were included. Principal component analysis (PCA) was firstly run on normalized methylation data to evaluate the similarity of methylation profile in 12 samples.

Bumphunter method was implemented to identify differentially methylated regions (DMR) within ChAMP (ChAMP.DMR function). The methylation beta value of each DMR was calculated using the mean value of all the probes that included in each DMR.

The heatmap was drawn using all the DMRs generated from ChAMP.DMR function. The |log2(FoldChange)| > 0.5 and Family-wise error rate (FWER) adjusted p-value < 0.05 was considered a statistical significance threshold. Therefore, the DMR regions with a |log2(FoldChange)| > 0.5 and FWER < 0.05 was considered as significantly differentially methylated regions. The DMRs region were annotated using ChIPseeker package in R bioconductor. The genomic regions for significantly differentially methylated regions were annotated with the definition of TSS3000 (3000 upstream and downstream of transcription initiation locus respectively) as the promoter region. Gene ontology (GO) functional enrichment analysis was done for the genes associated with differential methylation regions. And adjusted p-value < 0.05 was considered a statistical significance threshold.

### Quantitative reverse transcription PCR

Total RNA was extracted from ectopic and eutopic samples. cDNA was obtained after reverse transcription reversal by Evo M-MLV RT Kit (Accurate Biology Co. Ltd, AG11601). The qRT-PCR was performed by Evo M-MLV One Step RT-PCR Kit (Accurate Biology Co. Ltd, AG11607). Each target gene was compared to β-actin. The expression of target mRNA was calculated based on 2-ΔΔCt method.

### Statistical analysis

All the statistical analyses were performed using R software (version 4.0.1). P-values less than 0.05 were considered statistically significant. The Kolmogorov-Smirnov test was applied to evaluate the normality of the distribution of the variables. For qRT-PCR, statistical analyses were conducted using a student t-test (data with normal distribution) or Mann-Whitney test (data with skewed distribution) as appropriate by GraphPad Prism software 9.0 (GraphPad Software Inc).

## Results

### DNA methylation profiles showed the ovarian cysts were closely grouped with normal ovarian but not endometrial tissues

**Fig. 1:**
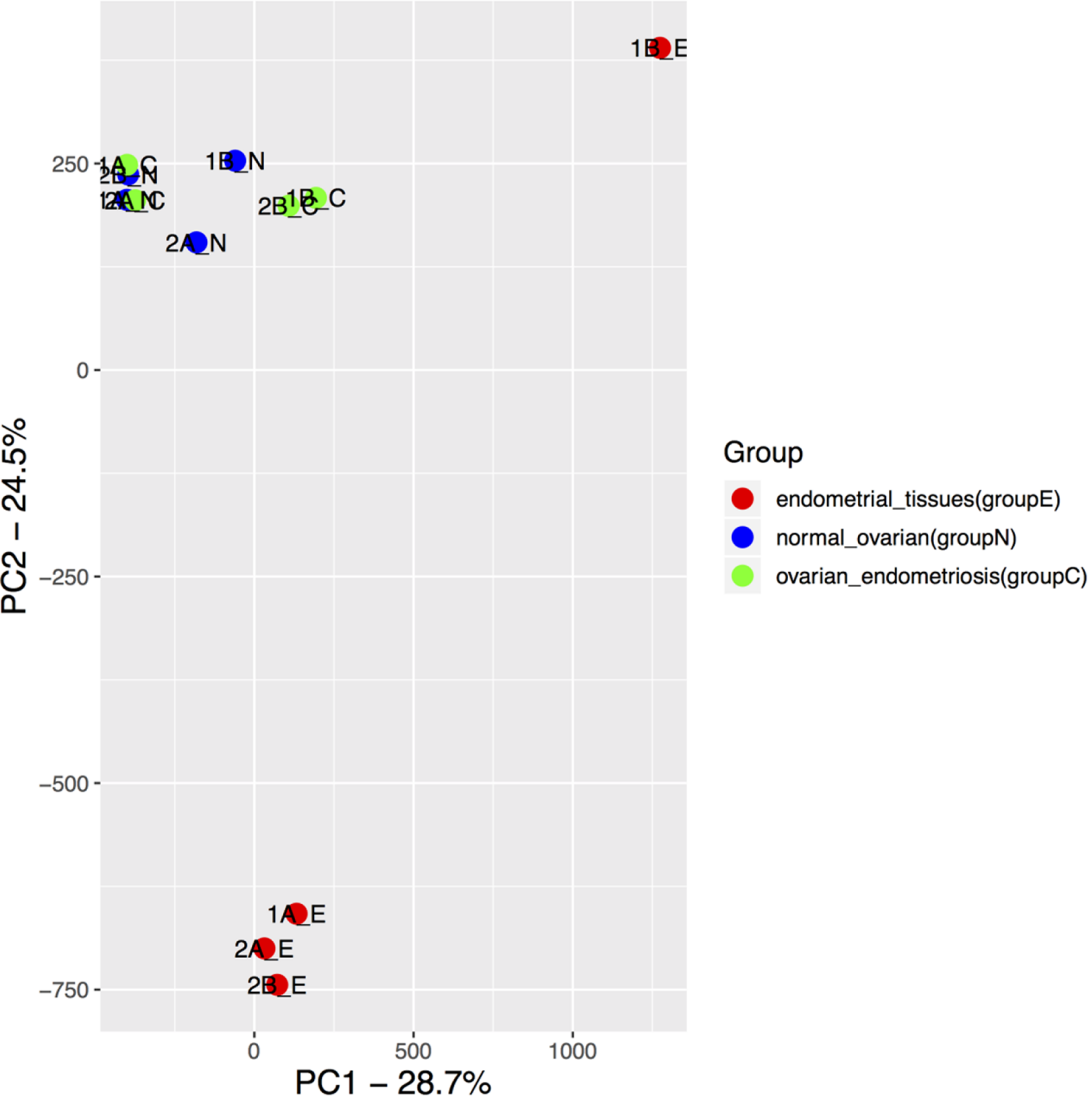
PCA plot of DNA methylation data from endometriosis, normal ovarian and endometrial tissues

**Fig. 2:**
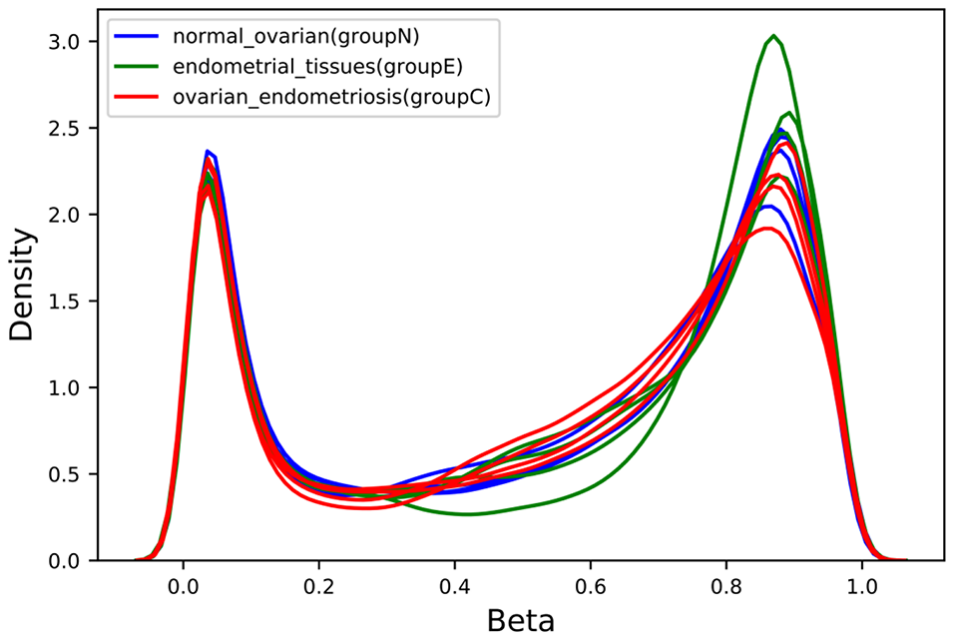
One of endometrial sample was located further away from other samples as an outlier, and this sample also showed a slightly different normalized beta distribution compared with other samples

**Fig. 3:**
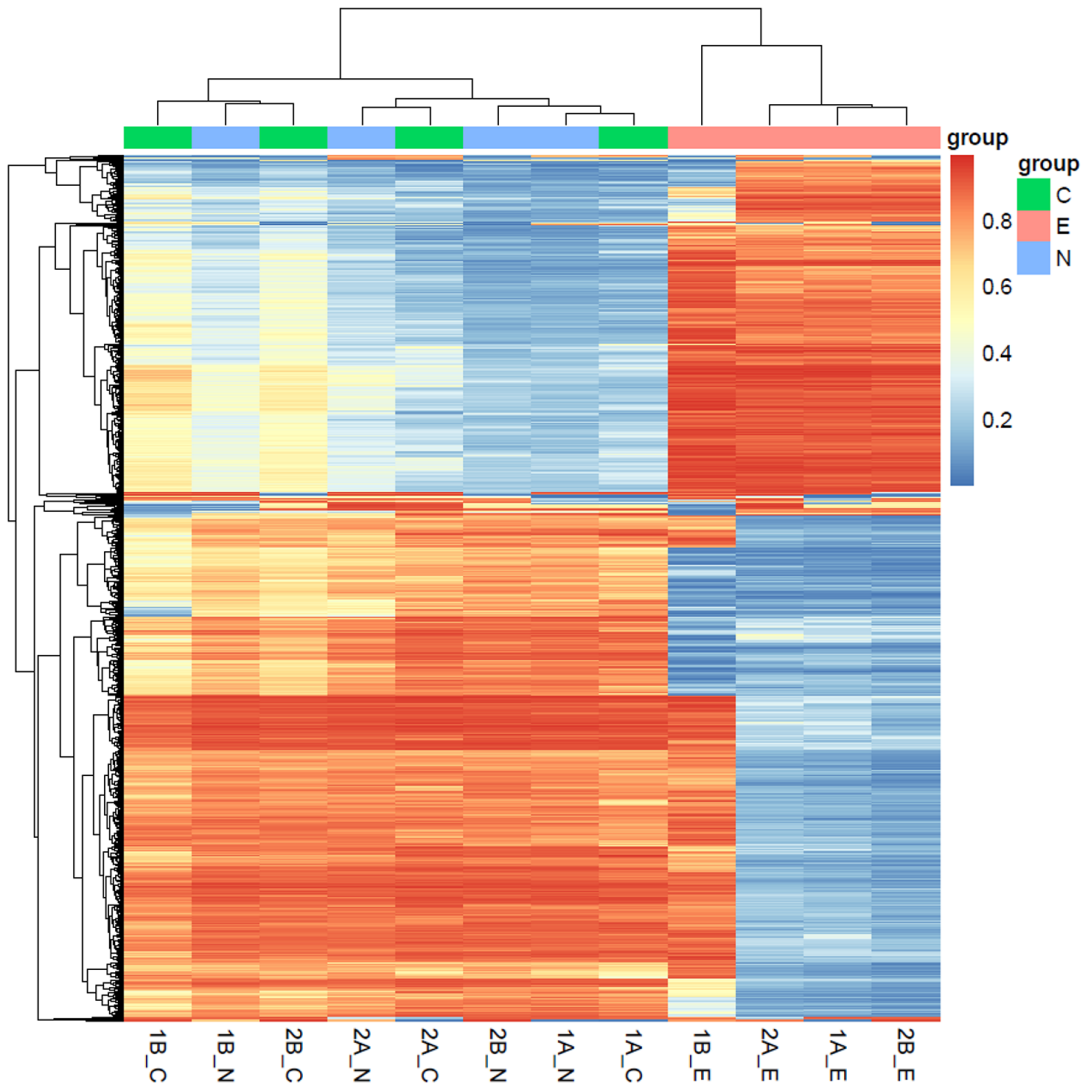
Hierarchical clustering analysis of 12 samples based on methylation levels. Top 1000 CpGs with the highest variance among 12 samples were included. Color mapping from blue to red indicates methylation level from low to high. Group C: ovarian endometrial cysts; N: normal_ovarian; E: eutopic endometriosis

**Fig. 4:**
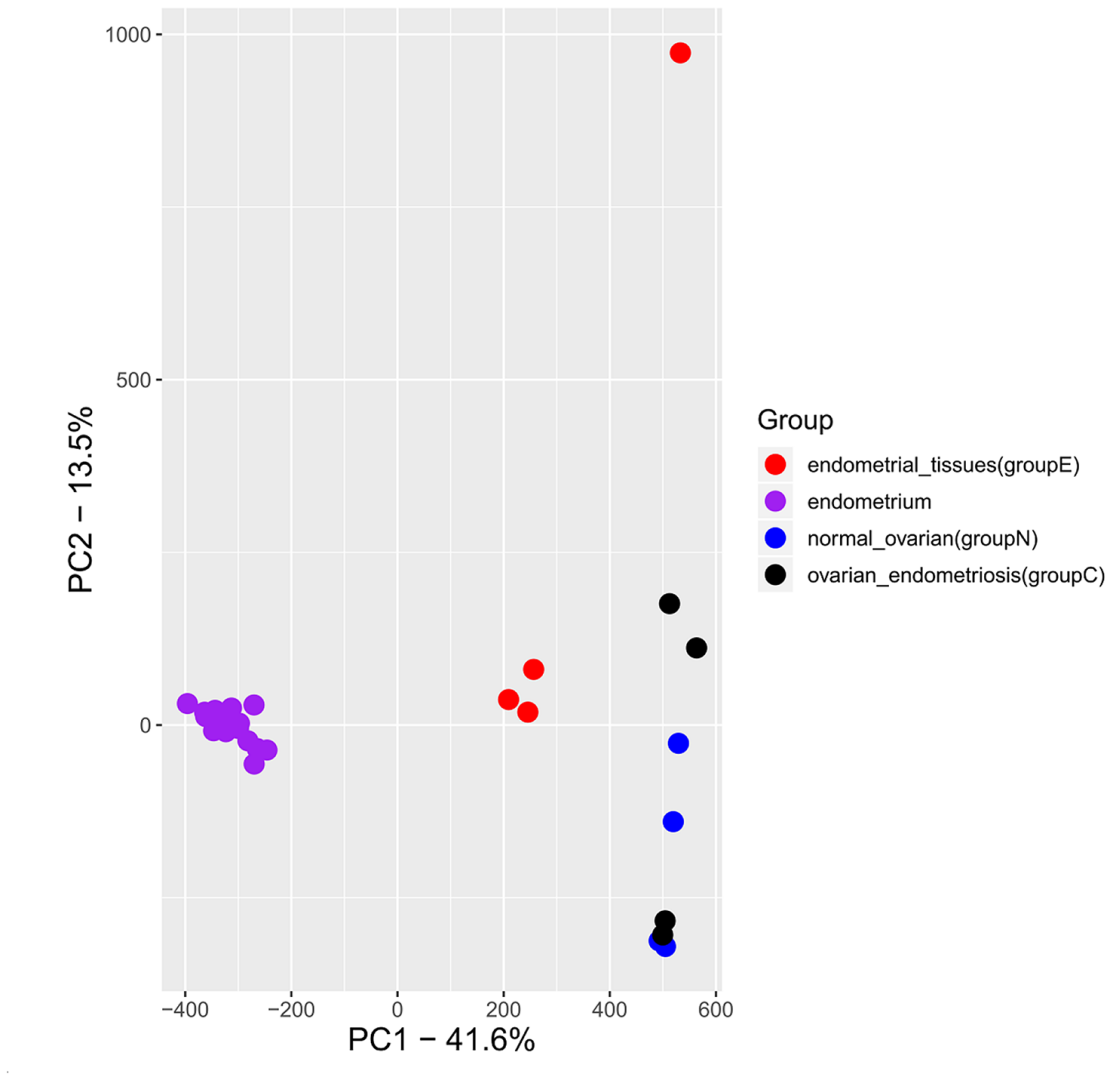
Endometriosis samples closely resembled normal ovarian tissues, but not endometrial tissues

Total of 12 samples from 4 patients, each with endometriosis, ovarian and endometrial tissues were profiled with illumina 850k methylation array. Principal component analysis clearly showed that endometriosis samples were tightly clustered with samples of ovarian tissue (Fig. 1). One of endometrial sample was located further away from other samples as an outlier, and this sample also showed a slightly different normalized beta distribution compared with other samples (Fig. 2). Then we plotted the heatmap of DNA methylation beta value matrix after normalization, and clustered the samples by hierarchical clustering. Again, 1B_E exhibited a hybrid DNA methylation profile.(Fig. 3) Therefore, we removed this sample for the downstream analyses. To confirm this result, we also performed PCA on our samples as well as publically available DNA methylation data on healthy endometrial biopsies [11], and as expected, healthy endometrial samples from public data were closer to our endometrial samples (Fig. 4).

**Fig. 5:**
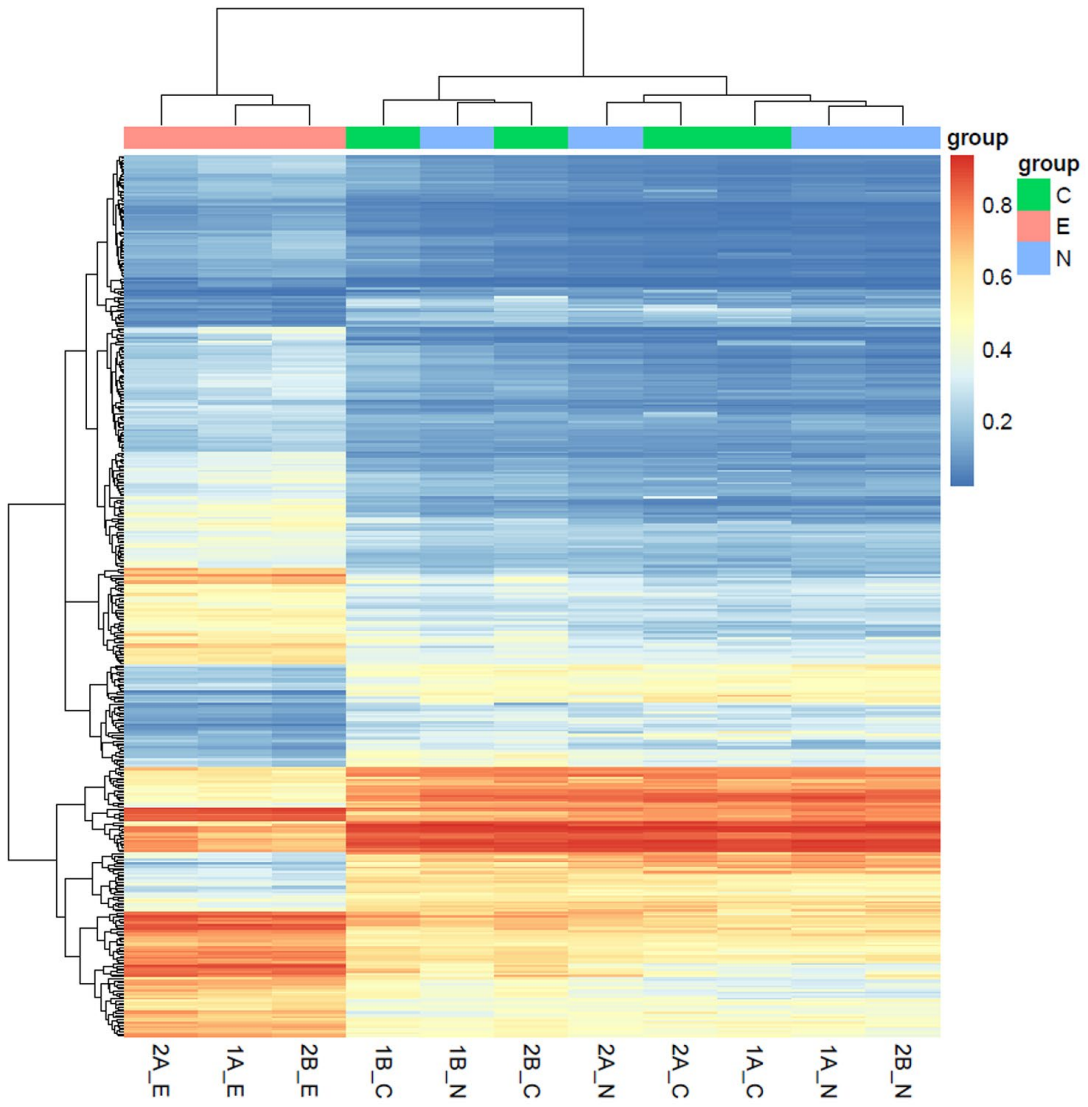
Hierarchical clustering analysis of the three groups based on DMRs. Color mapping from blue to red indicates methylation level from low to high. Group C: ovarian endometrial cysts; N: normal_ovarian; E: eutopic endometriosis

As for DMRs analysis, we used the software ChAMP to explore the DMRs and the methylation beta value of each DMR was calculated using the mean value of all the probes that included in each DMR. The heatmap was generated using the beta value of DMRs. And from the heatmap we can see the ovarian endometrial cysts (group C) and normal_ovarian (group N) are clusted together, suggesting that the methylation profile of the cyst is more similar to ovarian tissues compared with endometrial tissue (Fig. 5 ).

**Fig. 6:**
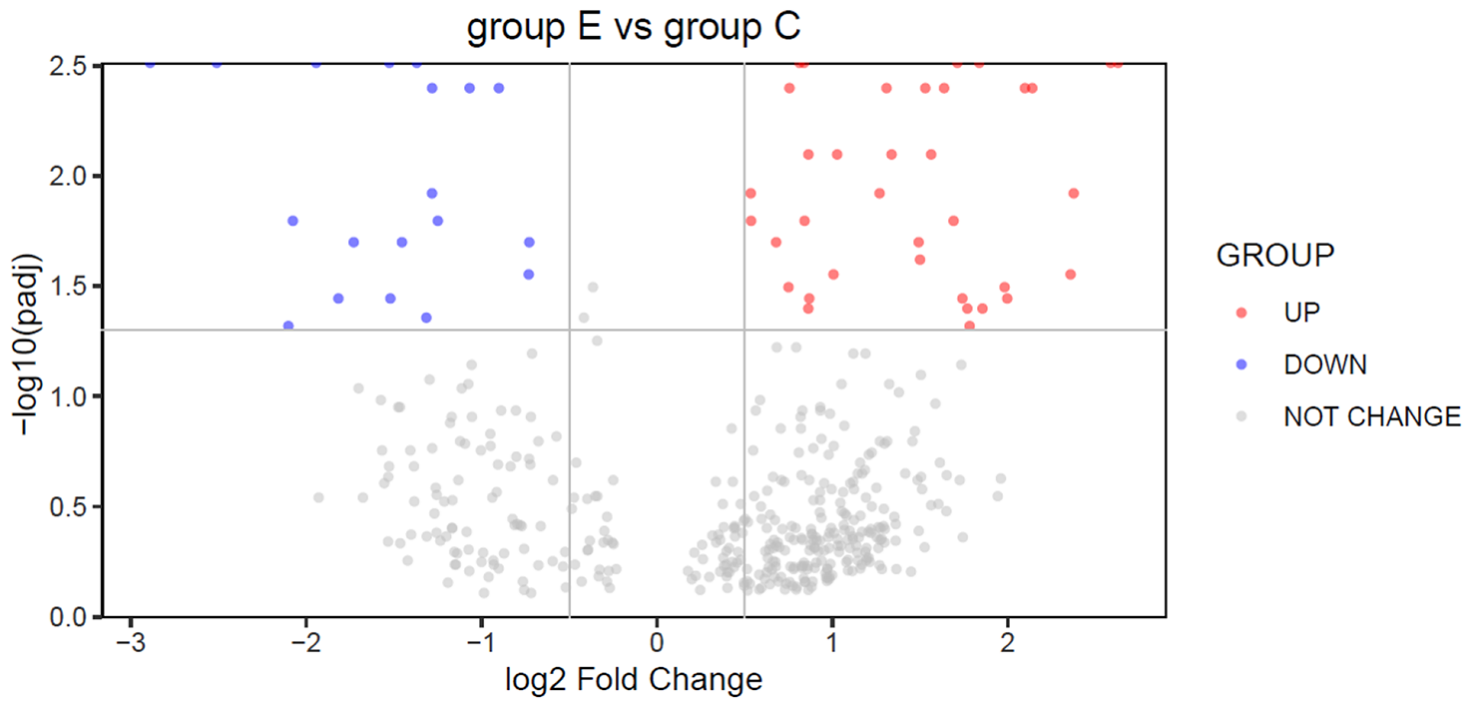
Volcano plot of log2(fold change) against ‑log10(p.adj ) of DMRs. The red blue spots stand for DMRs with adjusted p-value < 0.05 and log2FC > 0.5, and blue spots stand for DMRs with adjusted p-value < 0.05 and log2FC < -0.5, grey spots stand for non‑significant DMRs. The horizontal dash line denotes adjusted p-value < 0.05; the vertical dash lines denote |log2FC|>0.5. Group C: ovarian endometrial cysts; E: eutopic endometriosis

**Fig. 7:**
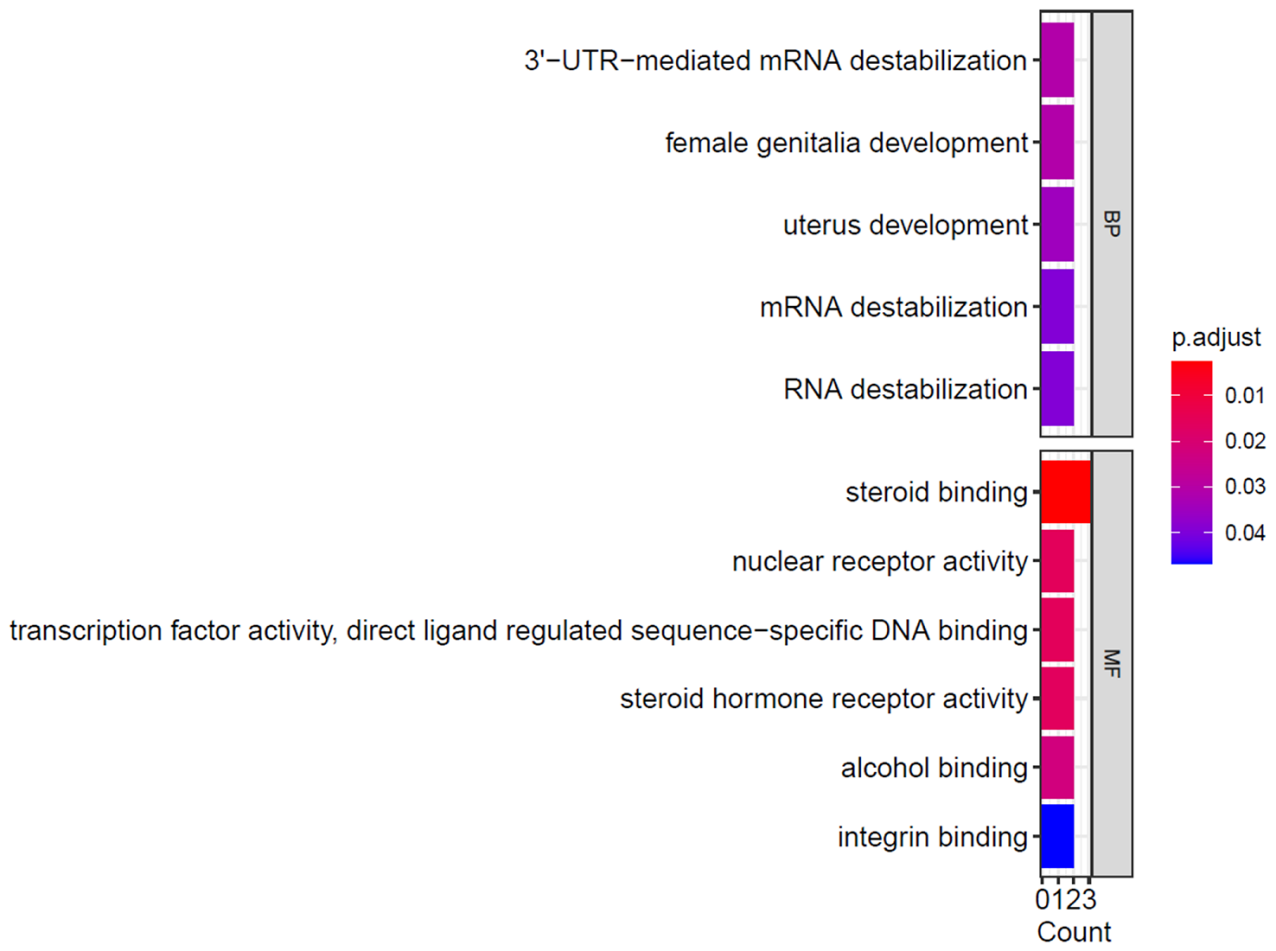
Significantly enriched term by the genes with higher methylation levels in ovarian endometrial cysts (group C). Genes with higher methylated levels in eutopic endometriosis (group E) compared with ovarian endometrial cysts (group C) GO analysis

**Fig. 8:**
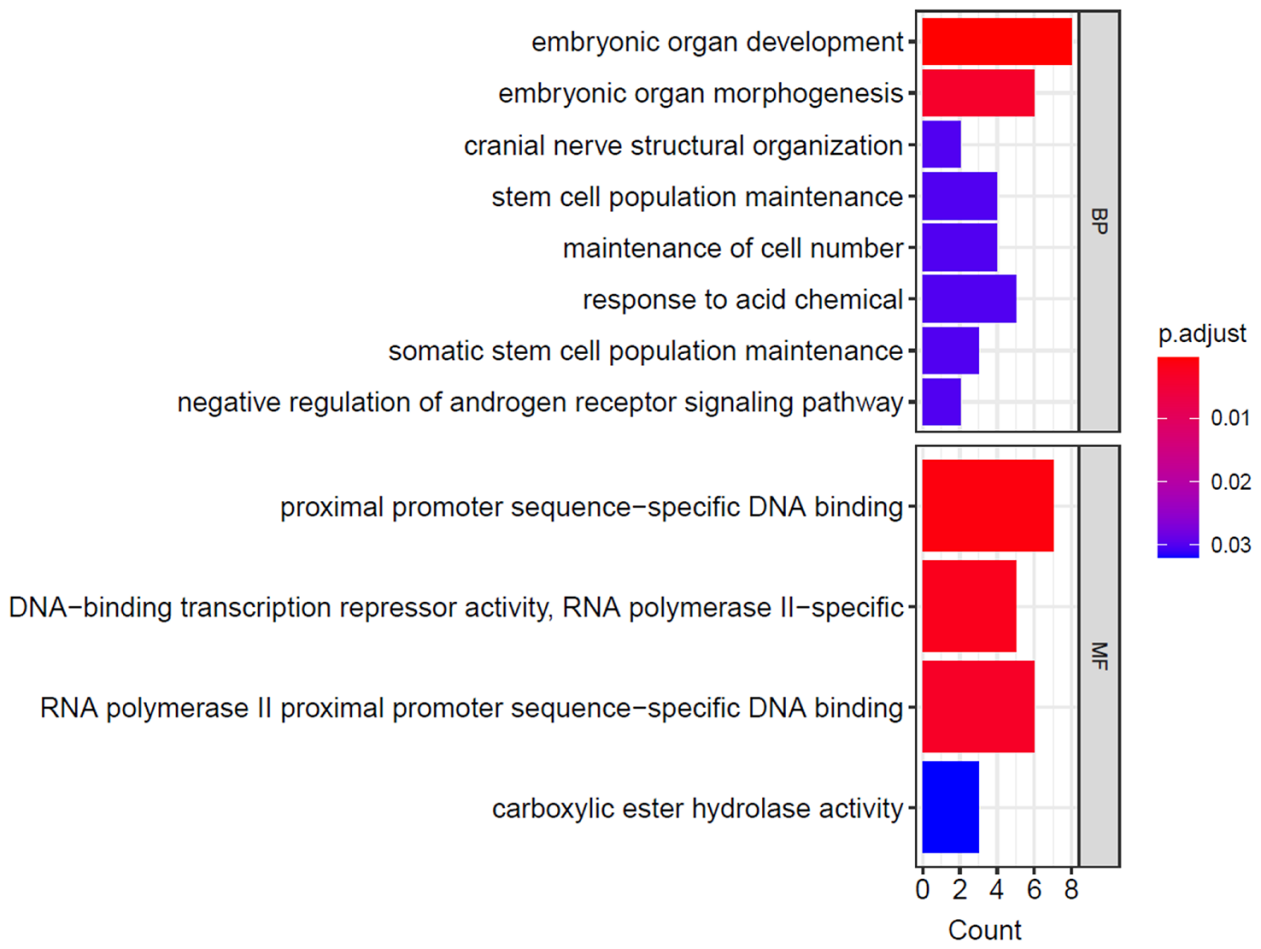
Significantly enriched term by the genes with lower methylation levels in ovarian endometrial cysts (group C)

We finally got 431 DMRs (with p-value < 0.05) using the champ.DMR function. But the counts of DMRs with |log2(FoldChange)| > 0.5 and adjusted p-value < 0.05 was 55 (Fig. 6). Figure 7 shows the GO analysis of Genes with lower methylation levels in eutopic endometriosis (group E) compared with in ovarian endometrial cysts (group C). And Fig. 8 shows the GO analysis of Genes with higher methylation levels in eutopic endometriosis (group E) compared with ovarian endometrial cysts (group C).

### Abnormal epigenetic profiles of genes involving receptors, signaling pathways and immune responses

Most of previous studies compared the endometriosis with endometrial tissues; therefore we analyzed differential DNA methylation between endometriosis and endometrial tissues and identified the differentially methylated regions (Table S1). In comparison with eutopic endometriosis, many genes in ovarian endometrial cysts (choESC) had different degrees of methylation, high and low (Tables 1 and 2),With acquired methylation profiles data, we identified the detailed features of aberrantly methylated genes in choESC using gene cards (the humor gene database) analyses. Depending on pathway analysis of screened genes, there are abnormalities of relevant signal transduction pathways involving endometriosis onset and progression, developmental processes, human early embryonic development, regulation of caspase, tyrosine, and mRNA metabolic processes et al. It indicated an abnormal expression pattern of choESC in peritoneal environment.

**Table 1:** Hypomethylation in endometrial tissues compared the endometriosis

Gene Symbol	Gene name	DMR value (Endometrial vs. Cyst)	Delta_beta	Log2 Fold Change Ratio	*P* value	Function
HOXB1	homeotic genes B1	3.749	0.386	0.813	0	Important growth and development regulatory genes regulator of retinoic acid response elements (RAREs)
MCC	membrane compartment of Can1	3.709	0.288	2.587	0	one cytoplasm membrane microdomain regulating metabolism, cellular morphogenesis, signaling cascades, and mRNA degradation.
TMEM101	transmembrane protein 101	3.695	0.297	2.629	0	upregulation and increase the expression of NF-κB in ovary cancer.
CASS4	Homo sapiens Cas scaffolding protein family member 4	3.624	0.515	1.530	0.004	Possible docking protein which may play a role for tyrosine-kinase-based signaling related to cell adhesion. Regulates FAK activity, focal adhesion integrity, and cell spreading.
BST2	bone marrow stromal cell antigen 2	3.612	0.450	1.713	0	Regulating Type-I interferon and interleukin-6 generation
FABP3	Fatty Acid Binding Protein 3	3.150	0.298	2.098	0.004	During the early stages of pregnancy, upregulating cell growth and proliferation in uterine endometrium
FAIM2	Fas Apoptotic Inhibitory Molecule	3.052	0.317	2.376	0.012	specifically protecting cells from Fas-induced apoptosis
SHANK2	SH3 and multiple ankyrin repeat domains protein	3.051	0.353	2.140	0.004	A protein-coding gene relating to autism
TCF21	Transcription Factor 21	3.030	0.433	1.563	0.008	augmenting steroidogenic factor (SF) 1 and estrogen receptor β (ERβ)
PRDM1	positive regulatory domain 1	2.983	0.431	1.269	0.012	increased IL-10 + Th17 cells are significantly
MIR365A	MicroRNA 365a	2.974	0.378	0.842	0.016	involving posttranscriptional modulation of genes via regulating mRNA stabilization and translation
FOXP1	Forkhead Box P1	2.849	0.443	1.492	0.02	activating Wnt/β-cateninsignaling pathway in endometriosis
LOC339803	LOC339803	2.841	0.199	2.358	0.028	Enhancing HCC invasion and migration via miR-30a-5p/ SNAIL1. In human atherosclerotic lesions, serve as potential biomarkers for lesion hypoxia.
ZNF311	Zinc Finger Protein311	2.833	0.330	1.838	0	upregulated in grade-depended glioma patients of adverse outcome
RNASE1	Ribonuclease A Family Member 1	2.773	0.364	1.027	0.008	a key player in regulation of vascular homeostasis
LYPLAL1	Long non coding RNA lysophospholipase -like l	2.771	0.206	1.338	0.008	Long non coding RNA lysophospholipase-like2
PAX2	Paired Box Homeotic Gene 2	2.670	0.291	1.741	0.036	Its mutations contribute to optical nerve glioma and kidney dysplasia
NUP210L	Nucleoporin 210	2.663	0.328	0.868	0.036	Encoding a membrane-spanning glycoprotein which participates in formation of nuclear pore complex
SLC1A2	Solute Carrier Family 1 Member 2	2.657	0.329	1.856	0.04	Encoding a solute transporter protein
MX2	MX Dynamin Like GTPase 2	2.646	0.252	1.770	0.04	Encoding a protein of dynamin and GTPases families, with nuclear and cytoplasmic forms.
SVIL	Supervillin	2.632	0.369	0.863	0.04	Encoded product contributes to myosin II assembly and disintegration of focal adhesions.
TERC	Telomerase RNA Component	2.614	0.227	1.500	0.024	Encoding telomerase.
PROCA1	Protein Interacting With Cyclin A1	2.588	0.259	1.982	0.032	Enables cyclin binding activity. Predicted to be involved in arachidonic acid secretion and phospholipid metabolic process
ARHGAP25	Rho GTPase Activating Protein 25	2.577	0.300	1.637	0.004	Encoding negative regulators of Rho GTPases.
DAW1	Dynein Assembly Factor With WD Repeats 1	2.569	0.226	0.679	0.02	Maybe the upstream of some processes, including cerebrospinal fluid circulation; determination of left/right symmetry; and outer dynein arm assembly.
MAGI2-AS3	MAGI2 Antisense RNA 3	2.563	0.264	1.783	0.048	An RNA Gene, affiliated with the lncRNA class. Diseases associated with MAGI2-AS3 include Nephrotic Syndrome, Type 15 and Breast Cancer.
TNFSF13B	TNF Superfamily Member 13b	2.488	0.179	2.000	0.036	modulating B cells proliferation and differentiation.
HOXA2	Homeobox A2	2.3473	0.330	0.864	0.008	Encoding a DNA-binding transcription factor governing morphogenesis, and differentiation.
SGIP1	SH3GL Interacting Endocytic Adaptor 1	2.103	0.196	1.691	0.016	May involve in clathrin-mediated endocytosis and energy homeostasis.
ASCL2	Achaete-Scute Family BHLH Transcription Factor 2	2.028	0.257	0.838	0	related pathways are Embryonic and Induced Pluripotent Stem Cells and Lineage-specific Markers and Human Early Embryo Development.
EPM2AIP1	EPM2A Interacting Protein 1	2.013	0.133	1.007	0.028	MLH1 and EPM2AIP1 genes share a common promoter whose methylation has been shown to affect both genes.
UBD	Ubiquitin D	1.818	0.140	1.309	0.004	Encoded protein participates in aggresomes formation, mitotic regulation, and dendritic cell maturation.
RNF39	RING finger protein 39	1.6484	0.201	0.535	0.012	Its variants were linked to viral diseases and autoimmune diseases.
TBX3	T-Box Transcription Factor 3	1.567	0.272	0.756	0.004	Regulating developmental processes.
PON1	Paraoxonase 1	1.390	0.180	0.537	0.016	Displaying lactonase and ester hydrolase activity.
ZIC1	Zic Family Member 1	1.157	0.138	0.750	0.032	Encoding a C2H2-type zinc finger proteins.

**Table 2:** Hypermethylation in endometrial tissues compared the endometriosis

Gene Symbol	Gene name	DMR value (Endometrial vs. Cyst)	Delta_beta	Log2 Fold Change Ratio	*P* value	Function
SHF	Src Homology 2 Domain Containing F	-2.107	-0,172	-1.249	0.016	May enable phosphotyrosine residue binding activation, and participate in apoptosis.
ESR1	Estrogen Receptor 1	-2.177	-0.296	-1.520	0.016	Encoding estrogen receptor, vital for hormone binding, and transcriptional activation.
ESR2	Estrogen Receptor 2	-2.280			0.036	Encoding the estrogen receptor 2.
RBM24	RNA Binding Motif Protein 24	-2.414	-0.249	-1.816	0.036	Involved in several processes, including negative regulation of cytoplasmic translation, and regulation of mRNA metabolic process.
LMO7DN	LIM Domain 7 downstream neighbour	-2.487	-0.334	-0.728	0.02	Involved in lung cancer prognosis.
ZC3H12D	Zinc Finger CCCH-Type Containing 12D	-2.541	-0.327	-1.454	0.02	Predicted to enable endoribonuclease activity and mRNA binding activity, and negatively modulate G1/S transition and cell growth
CACNB2	Calcium Voltage-Gated Channel Auxiliary Subunit Beta 2	-2.550	-0.234	-1.729	0.02	Encoding a subunit of voltage-dependent calcium channel proteins.
C17orf107(CHRNE)	Cholinergic Receptor Nicotinic Epsilon Subunit	-2.563	-0.197	-2.101	0.048	After binding acetylcholine, AChR undergoes an alteration in conformation opening an ionotransduction channels across the plasma membrane
HAND2-AS1	HAND2 Antisense RNA 1	-2.605	-0.383	-1.315	0.044	Predicted to be involved in positive regulation of gene expression, and possess a positive effect on cardiac right ventricle morphogenesis.
PEMT	Phosphatidylethanolamine N- Methyltransferase	-2.800	-0.346	-0.731	0.028	Converting phosphatidylethanolamine to phosphatidylcholine by sequential methylation.
ZNF22	Zinc Finger Protein22	-2.837	-0.259	-1.282	0.012	Modulating cell migration, adhesion, and cycle.
STRA6	Signaling Receptor And Transporter Of Retinol STRA6	-3.033	-0.430	-1.369	0	Encoding a membrane protein responsible for retinol metabolism.
LINC00460	Long Intergenic Non-Protein Coding RNA 460	-3.090	-0.351	-1.068	0.004	play vital roles in the pathogenesis, tumorigenesis, and angiogenesis of cancers.
LRMDA	Leucine Rich Melanocyte Differentiation Associated	-3.270	-0.442	-1.282	0.004	This gene encodes a leucine-rich repeat protein.
EMX2OS	EMX2OS	-3.272	-0.445	-1.943	0	Modulating ovarian cancer cells through miR-654-3p/AKT3/PD-L1.
RNF19A	RING finger protein 19 A	-3.461	-0.448	-1.526	0	Interacting with alpha synuclein in neurons.
HOXC4	Homeobox C4	-3.463	-0.435	-2.510	0	Related to immunodeficiency with Hyper-Igm, and lymphoma.
TFAMP1	Transcription Factor A, Mitochondrial Pseudogene 1	-3.641	-0.391	-0.901	0.004	Encoding a mitochondrial transcription factor.
FMN1	Formin 1	-4.035	-0.407	-2.890	0	Modulating development of adhesion junction and linear actin polymerization.

GO term enrichment analysis showed that the hypomethylation genes in ovarian endometrial cysts were primarily engaged in embryonic organ development and embryonic organ development, stem cell population maintenance, et al. And the hypermethylation genes in ovarian endometrial cysts were primarily engaged in mRNA destabilization, female uterus and genitalia development, et al. The function of genes related to steroid binding, transcription factor activity and receptor activity.(Figures 7 and 8 ).

**Fig. 9:**
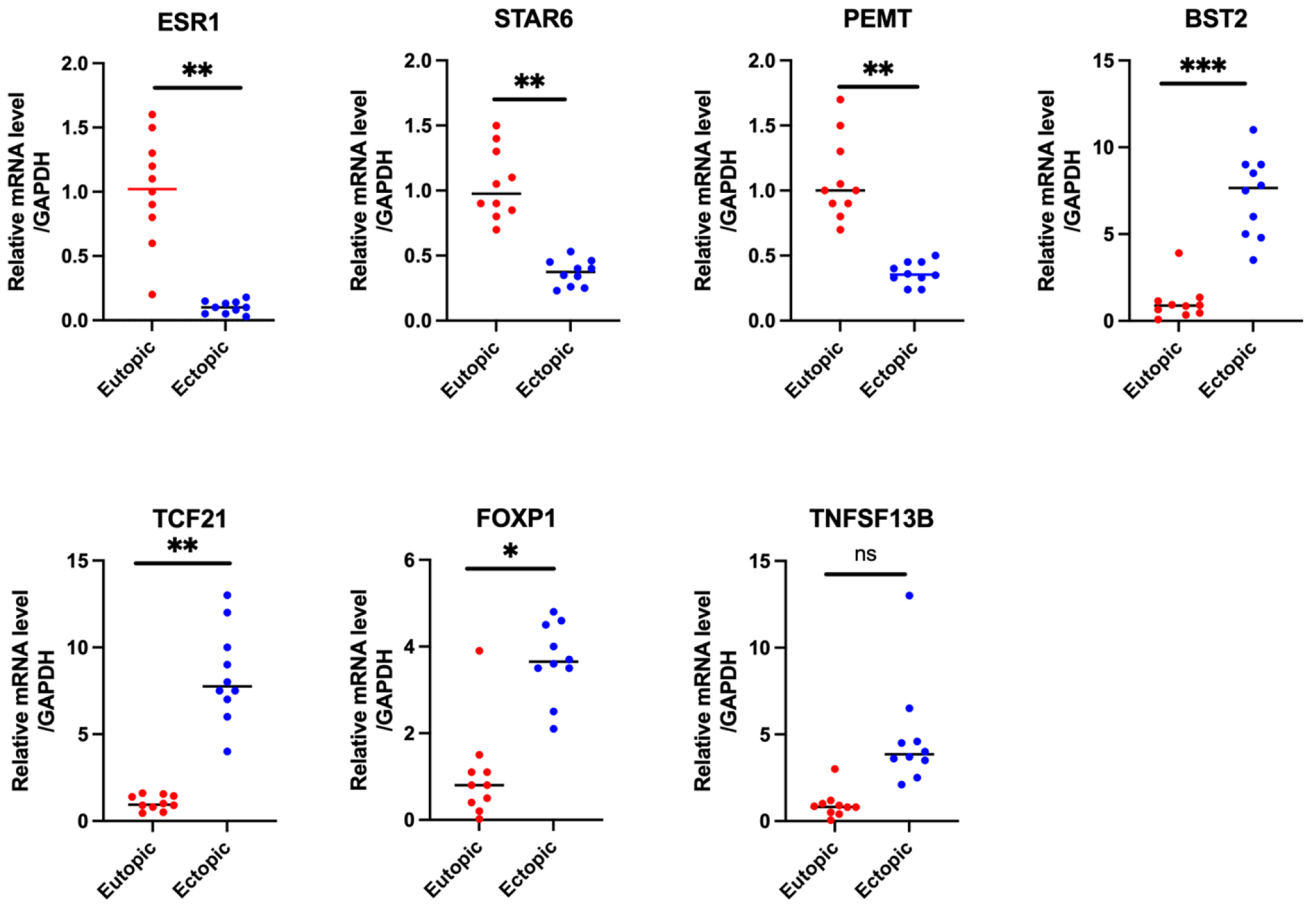
qRT-PCR validation of the gene expression of the candidate genes. ER1,STAR6 and PEMTwere significantly downregulated in ectopic tissues, and BST2,TCF21 and FOXP1 were significantly upregulated in ectopic tissues. ***P < 0.001, **P < 0.01, *P < 0.05, ns, not significant

We compared our differentially methylated sites and only found consistent results on hyper-methylation on ESR1 [1, 12].Consistently, downregulation of ESR1 mRNA level in endometriosis was also reported in few studies [12, 13]. Then we searched for the literature for the differentially methylated genes we identified and examined whether the associated expression patterns were observed. Indeed, we observed consistent results on 5 genes: TNFSF13B, FOXP1, TCF21, BST2 and STRA6 [14–20].Taken together, despite little overlap with previously characterized genes, the characterized methylated genes in endometriosis were consistent with the reported expression changes in endometriosis(Table 3).RT-PCR results showed that ER1,STAR6 and PEMT were significantly downregulated in ectopic tissues, and BST2,TCF21 and FOXP1 were significantly upregulated in ectopic tissues (Fig. 9 ). In summary, we validated the top candidate genes in endometriosis which might be regulated by DNA methylation.

**Table 3:** The differentially methylated genes were consistent with the reported expression changes in endometriosis

Gene Symbol	Gene name	DMR value (Endometrial vs. Cyst)	Delta_beta		Log2 Fold Change Ratio		*P* value		Function		
Hypomethylation											
BST2	bone marrow stromal cell antigen 2	3.612	0.450		1.713		0		Regulating type-I interferon and interleukin-6 generation Modulating embryo-maternal immune.		
TCF21	Transcription Factor 21	3.030	0.433		1.563		0.008		Interacting with upstream stimulatory factor 2, transactivating SF-1 and Erβ promoters, and modulating estrogen pathway and fibrosis in endometriosis.		
FOXP1	Forkhead Box P1	2.849	0.443		1.492		0.02		FOXP1 activating Wnt/β-cateninsignaling pathway in endometriosis.Its knockdown reverted endometrium cell phenotypes.		
TNFSF13B	TNF Superfamily Member 13b	2.488	0.179		2.000		0.036		Modulating B cells proliferation and differentiation. Increased in serum of endometriosis subjects.		
Hypermethylation											
ESR1	Estrogen Receptor 1	-2.177	-0.296		-1.520		0.016		Encoding estrogen receptor, vital for hormone binding, and transcriptional activation.		
STRA6	Signaling Receptor And Transporter Of Retinol STRA6	-3.033	-0.430		-1.369		0		Vital for retinol binding protein, the retinol uptake into cells. Downregulating STRA6 enhanced endogenous estradiol synthesis.		
PEMT	Phosphatidylethanolamine N- Methyltransferase	-2.800		-0.259		-1.282		0.028		Converting phosphatidylethanolamine to phosphatidylcholine by sequential methylation. Its polymorphism may induce infertility of endometriosis women.	

## Discussion

In this work, we presented a very small cohort of paired DNA methylation analysis of normal ovarian, endometrial and endometriosis samples. Despite the very small sample size, results were very clear that endometriosis samples closely resembled normal ovarian tissues, but not endometrial tissues. Such results suggested that, instead of migrating from endometrial tissues, endometriosis might be originated from ovarian tissues. Such results did not rule out the possibility that endometriosis was indeed seeded by endometrial cells, but drastic DNA methylation reprogramming resulted in highly similar DNA methylation profiles to the ovarian tissues.

A recent report confirmed genome-wide DNA methylation profiles of endometriosis tissue subtypes, focusing on promoters [21]. They found a large number of methylation differences compared with the eutopic endometrium and verified with clinical data. At the same time, Yoshiaki et al. revealed a more accurate genome-wide DNA methylation map, which was derived from the homogenous ESCs of eutopic endometrium and ovarian endometrium cysts with endometriosis. Some differentially methylated or expressed genes (Nr5a1, star, STRA6 and HSD17B2) were involved in steroidogenesis, also verified in numerous clinical specimens by independent methods [20]. Our strategy was to investigate whether there were methylation differences between multilocular ovarian cysts and unilocular ovarian cysts and normal ovarian tissues.

Many genes in choESC acquired from endometrium cysts had different degrees of methylation. Basing on acquired methylation profiles, specific features of aberrantly methylated genes in choESC were evaluated using gene cards (the humor gene database) analysis. There are abnormalities of relevant signal transduction pathways in endometriosis pathogenesis and progression. Abnormal methylation status exists in genes related to proliferation and apoptosis, and immune responses, indicating an abnormal expression of choESC in peritoneal environment. Meanwhile, choesc showed abnormal differentiation, such as neurogenesis and embryogenesis. This means that choesc may have differentiated into other types of cells, suggesting the existence of abnormal developmental processes. The above findings add the possible involvement of DNA epigenetic modifications in endometriosis pathogenesis.

We compared our differentially methylated sites and only found consistent results on hyper-methylation on ESR1. Consistently, downregulation of ESR1 mRNA level in endometriosis was also reported in few studies. Then we searched for the literature for the differentially methylated genes we identified and examined whether the associated expression patterns were observed. Indeed, we observed consistent results on 5 genes: TNFSF13B, FOXP1, TCF21, BST2 PEMT and STRA6. Increased TNFSF13B B lymphocyte stimulator protein was identified in serum of endometriosis subjects [14]. Suppressing FOXP1 reverted the endometrium cell phenotype, involving decreased collagen gel contraction, cell growth and migratory movement [15].TCF21 could transactivate SF-1 and Erβ promoters in ESCs [16], modulating estrogen pathway and fibrosis of endometriosis [17]. rs4244593 of PEMT-related polymorphism modulated the choline or phospholipids generation, inducing infertility of endometriosis women [19].STRA6 is vital for retinol binding protein, and retinol uptake into cells.

The correlation analysis between DNA methylation and transcriptomes identified an anticipated positive relation, as well as a negative relation. It is impossible to characterize the close relationship between DNA methylation and mRNA expression due to the lesser gene coverage of beadchip specifications and the analysis of only two CPGs per gene. In genome-wide observation, a complex network may exist between DNA methylation and transcription.

Altogether, this study provides basic DNA methylation data on ovarian endometriosis compared with ovarian tissue and eutopic endometrium using untreated cultured ESCs. We believe that this kind of new information will contribute to the future research on treatment strategies and preventive drugs for endometriosis.

### Electronic supplementary material

Below is the link to the electronic supplementary material.

Supplementary Material 1

## Data Availability

No datasets were generated or analysed during the current study.

## References

[1] Wang Y, Nicholes K, Shih IM (2020). The origin and Pathogenesis of endometriosis. Annu Rev Pathol.

[2] Yuan Z, Wang L, Wang Y, Zhang T, Li L, Cragun JM (2014). Tubal origin of ovarian endometriosis. Mod Pathol.

[3] Fraunhoffer NA, Meilerman Abuelafia A, Stella I, Galliano S, Barrios M, Vitullo AD (2015). Identification of germ cell-specific VASA and IFITM3 proteins in human ovarian endometriosis. J Ovarian Res.

[4] Bulun SE, Endometriosis (2009). N Engl J Med.

[5] Kao LC, Germeyer A, Tulac S, Lobo S, Yang JP, Taylor RN (2003). Expression profiling of endometrium from women with endometriosis reveals candidate genes for disease-based implantation failure and infertility. Endocrinology.

[6] Burney RO, Talbi S, Hamilton AE, Vo KC, Nyegaard M, Nezhat CR (2007). Gene expression analysis of endometrium reveals progesterone resistance and candidate susceptibility genes in women with endometriosis. Endocrinology.

[7] Wu Y, Halverson G, Basir Z, Strawn E, Yan P, Guo SW (2005). Aberrant methylation at HOXA10 may be responsible for its aberrant expression in the endometrium of patients with endometriosis. Am J Obstet Gynecol.

[8] Xue Q, Lin Z, Yin P, Milad MP, Cheng YH, Confino E (2007). Transcriptional activation of steroidogenic factor-1 by hypomethylation of the 5’ CpG island in endometriosis. J Clin Endocrinol Metab.

[9] Izawa M, Harada T, Taniguchi F, Ohama Y, Takenaka Y, Terakawa N (2008). An epigenetic disorder may cause aberrant expression of aromatase gene in endometriotic stromal cells. Fertil Steril.

[10] Tian Y, Morris TJ, Webster AP, Yang Z, Beck S, Feber A (2017). ChAMP: updated methylation analysis pipeline for Illumina BeadChips. Bioinformatics.

[11] Kukushkina V, Modhukur V, Suhorutšenko M, Peters M, Mägi R, Rahmioglu N (2017). DNA methylation changes in endometrium and correlation with gene expression during the transition from pre-receptive to receptive phase. Sci Rep.

[12] Maekawa R, Mihara Y, Sato S, Okada M, Tamura I, Shinagawa M (2019). Aberrant DNA methylation suppresses expression of estrogen receptor 1 (ESR1) in ovarian endometrioma. J Ovarian Res.

[13] Xue Q, Lin Z, Cheng YH, Huang CC, Marsh E, Yin P (2007). Promoter methylation regulates estrogen receptor 2 in human endometrium and endometriosis. Biol Reprod.

[14] Christofolini DM, Cavalheiro CM, Teles JS, Lerner TG, Brandes A, Bianco B (2011). Promoter – 817C > T variant of B lymphocyte stimulator gene (BLyS) and susceptibility to endometriosis-related infertility and idiopathic infertility in Brazilian population. Scand J Immunol.

[15] Shao X, Wei X (2018). FOXP1 enhances fibrosis via activating Wnt/β-catenin signaling pathway in endometriosis. Am J Transl Res.

[16] Wu PL, Zhou Y, Zeng C, Li X, Dong ZT, Zhou YF (2018). Transcription factor 21 regulates expression of ERβ and SF-1 via upstream stimulatory factor-2 in endometriotic tissues. Biochim Biophys Acta Gene Regul Mech.

[17] Ganieva U, Nakamura T, Osuka S, Bayasula, Nakanishi N, Kasahara Y (2020). Involvement of Transcription Factor 21 in the Pathogenesis of fibrosis in endometriosis. Am J Pathol.

[18] Vestergaard AL, Knudsen UB, Munk T, Rosbach H, Martensen PM (2011). Transcriptional expression of type-I interferon response genes and stability of housekeeping genes in the human endometrium and endometriosis. Mol Hum Reprod.

[19] Szczepańska M, Mostowska A, Wirstlein P, Lianeri M, Marianowski P, Skrzypczak J (2011). Polymorphic variants of folate and choline metabolism genes and the risk of endometriosis-associated infertility. Eur J Obstet Gynecol Reprod Biol.

[20] Yamagata Y, Nishino K, Takaki E, Sato S, Maekawa R, Nakai A (2014). Genome-wide DNA methylation profiling in cultured eutopic and ectopic endometrial stromal cells. PLoS ONE.

[21] Borghese B, Barbaux S, Mondon F, Santulli P, Pierre G, Vinci G (2010). Research resource: genome-wide profiling of methylated promoters in endometriosis reveals a subtelomeric location of hypermethylation. Mol Endocrinol.

